# Role of the microtubules in the electrical activity of the primary cilium of renal epithelial cells

**DOI:** 10.3389/fmolb.2023.1214532

**Published:** 2023-11-22

**Authors:** Noelia Scarinci, Brenda C. Gutierrez, Virginia H. Albarracín, María del Rocío Cantero, Horacio F. Cantiello

**Affiliations:** ^1^ Laboratorio de Canales Iónicos, Instituto Multidisciplinario de Salud, Tecnología y Desarrollo, IMSaTeD (UNSE-CONICET), Santiago del Estero, Argentina; ^2^ Centro Integral de Microscopía Electrónica (CIME-CONICET-UNT), Tucumán, Argentina

**Keywords:** primary cilia, electrical oscillations, electrical antenna, polycystin-2, microtubules, axoneme

## Abstract

The primary cilium is a non-motile sensory organelle that transduces environmental cues into cellular responses. It comprises an axoneme, a core of nine doublet microtubules (MTs) coated by a specialized membrane populated by receptors, and a high density of ion channels. Dysfunctional primary cilia generate the pathogenesis of several diseases known as ciliopathies. However, the electrical role of MTs in ciliary signaling remains largely unknown. Herein, we determined by the patch clamp technique the electrical activity of cytoplasmic and axonemal MTs from wild-type LLC-PK1 renal epithelial cells. We observed electrical oscillations with fundamental frequencies at ∼39 Hz and ∼93 Hz in sheets of cytoplasmic MTs. We also studied *in situ* and isolated, intact and Triton X-permeabilized primary cilia, observing electrical oscillations with peak frequencies at either 29–49 Hz (non-permeabilized) or ∼40–49 Hz (permeabilized) and ∼93 Hz (both). We applied Empirical Mode Decomposition (EMD), Continuous Wavelet Transform (CWT), and Cross-Correlation Analysis (CCA) to assess the differences and the coherence in the Time-Frequency domains of electrical oscillations between cytoplasmic and axonemal MTs. The data indicate that axonemal and cytoplasmic MTs show different patterns of electrical oscillations preserving coherence at specific frequency peaks that may serve as electromagnetic communication between compartments. Further, the electrical behavior of axonemal MTs was modified by siRNA deletion of polycystin-2 (PC2), which lengthens primary cilia, thus linking ciliary channels to the morphological and electrical behavior of cilia in ciliopathies. The encompassed evidence indicates that the primary cilium behaves as an electrical antenna, with an excitable MT structure that produces electrical oscillations whose synchronization and propagation constitute a novel cell signaling mechanism.

## 1 Introduction

The primary cilium is a sensory organelle that protrudes from the center of most eukaryotic cells, particularly renal epithelial cells (Wheatley, 1995). Motile cilia, also known as flagella, distinguish from primary cilia because of their pattern of ciliary MTs. The axoneme of motile cilia comprises nine doublets of peripheral MTs surrounding two central MTs (9 + 2) (Wheatley, 1995). Primary cilia lack the central MTs and have a 9 + 0 pattern. The pattern of doublet MTs observed in the axoneme of cilia and flagella is unique to these organelles. Changes in shape and the loss of motility facilitated the diversification of ciliary function into sensory properties. Primary cilia, such as those found in the brain or the olfactory epithelium, are typically rod or whip-shaped.

In contrast, other specialized cilia, such as those found in vertebrate rods and cone photoreceptors, have elaborate distal ciliary segments. Cilia are relevant to eukaryotic cell homeostasis and development, tissue physiology, and a continuously-expanding number of human disorders known as ciliopathies, such as autosomal dominant polycystic kidney disease (ADPKD) (Afzelius, 2004; Bisgrove and Yost, 2006; Quinlan et al., 2008; Carrascosa Romero et al., 2011; Hildebrandt et al., 2011). Primary cilia are surrounded by a continuous plasma membrane, the ciliary membrane, with a unique endowment of proteins, including ion channels (Nauli et al., 2003; Raychowdhury et al., 2005) and receptors (Handel et al., 1999; Brailov et al., 2000; Masyuk et al., 2008; Milenkovic et al., 2009; Raychowdhury et al., 2009). The primary cilium morphology extends to the basal body, the base of the axoneme MT doublets, and the centriole in a way that is coordinated with the cell cycle (Plotnikova et al., 2009). The core of the primary cilium is its MT-based axoneme, which is present in most vertebrate cell types, and conserved in most extant protists (Mitchell, 2007; Carvalho-Santos et al., 2011). Contrary to our understanding of cilia and flagella’s morphological and biological features, there needs to be more knowledge regarding axonemal MTs’ role in these organelles’ sensory functions.

MTs are highly charged hollow cylinders assembled from protofilaments of αβ-tubulin dimers (Dustin, 1984; Desai and Mitchison, 1997; Amos, 2004). The MT surface assembles into different lattices by lateral apposition of protofilaments (Freedman et al., 2009; Freedman et al., 2010). MTs are nonlinear electrical transmission lines (Priel et al., 2006; Priel et al., 2008; Priel and Tuszyński, 2008; Sekulić et al., 2011; Sekulić and Satarić, 2012). Previous studies from our laboratory showed that different mammalian brain MT structures, including 2D sheets and bundles, generated strong electrical oscillations (Cantero et al., 2016; Cantero et al., 2018). This electrical behavior is also found in isolated MTs (Gutierrez et al., 2023). This property of MTs is mechanistically consistent with that of organic electrochemical transistors that support both amplification and self-sustained current- (and voltage-) oscillations (Cantero et al., 2016; Cantero et al., 2018; Cantero and Cantiello, 2020; Cantero et al., 2020; Gutierrez et al., 2023). MTs display properties of memristive devices (Cantero et al., 2019), and their assemblies show the capability of evolutionary computation similar to that observed in assemblies of nanotubes (Vissol-Gaudin et al., 2021). Thus, we posed the idea that the electrical activity of axonemal MTs may be at the center of the sensory properties of cilia and flagella.

**FIGURE 1:**
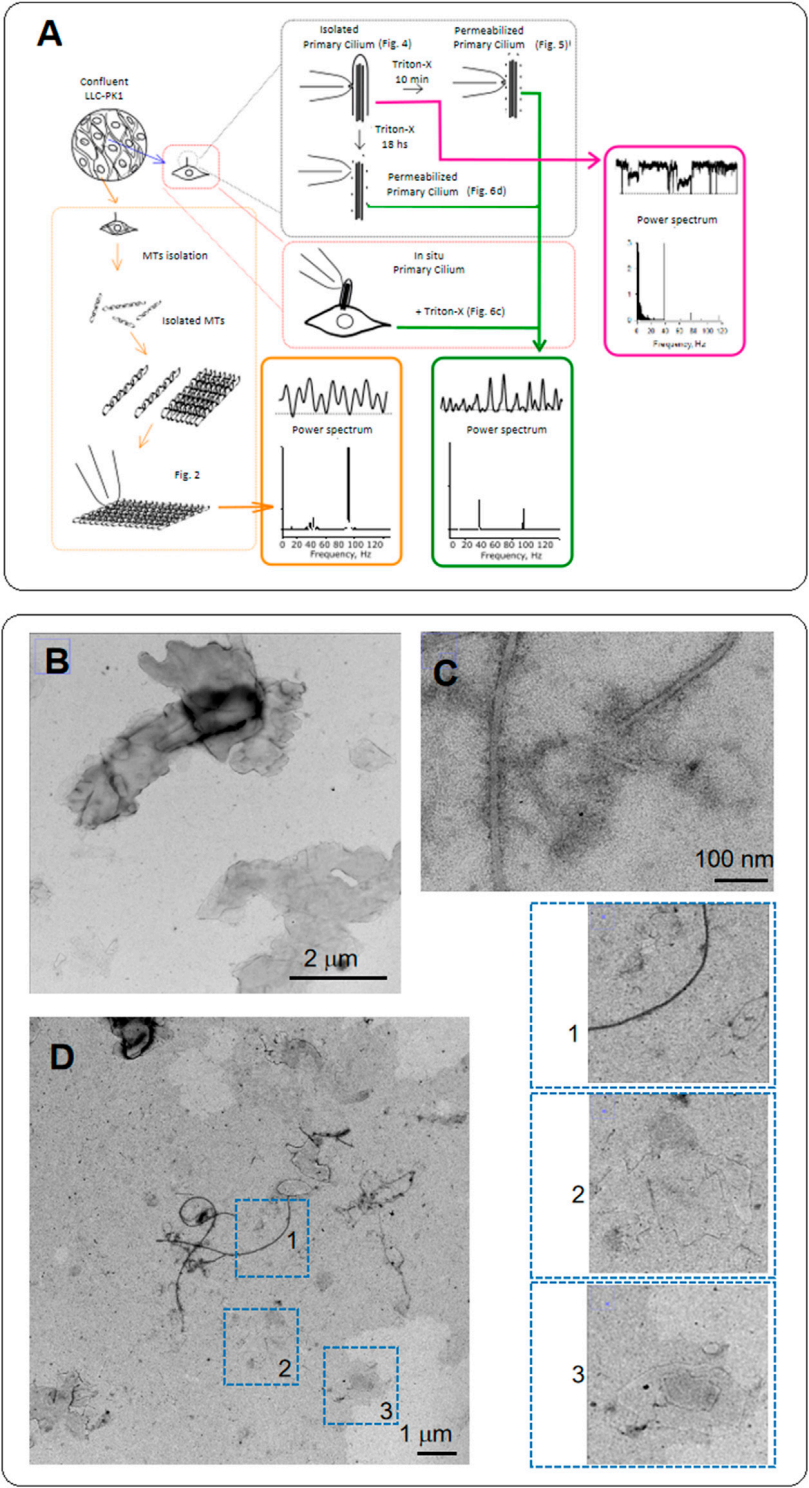
Diagram of experiments and electron microscopy of MTs from LLC-PK1 renal epithelial cells. **(A)** Confluent monolayers of LLC-PK1 cells were used to isolate and purify cytoplasmic MTs and obtain 2D sheets. We isolated primary cilia and patched the ciliary membrane to obtain channel recordings. The ciliary membrane was subsequently permeabilized to record electrical data from the axoneme. We further Triton X-permeabilized intact cells to obtain electrical recordings of primary cilia *in situ*. We finally conducted Time-Frequency and coherence analyses to detect waveform differences between the cytoplasmic and axonemal MTs. **(B)** Negative staining of MT sheets from LLC-PK1 cells. The grayscale identifies the height of the sheets stacked on each other. **(C)** Negative staining of isolated MTs in the MT sheet preparation. The thickness of the longer ones is about 25 nm. However, flat MTs are also observed where individual protofilaments, including a smaller MT-like structure, can be identified. **(D)** Negative staining of the LLC-PK1 preparation showing MT sheets. Insets on the Right shows isolated MTs (1), a small MT sheet (2), and at least two MT sheets superposed (3). Small MT sheets indicate the arrangement of parallel stacks of protofilaments. Small sheets are thinner than the expected MT size.

To gain insight into the electrical behavior of ciliary MTs, we explored the presence of electrical oscillations in primary cilia obtained from a non-excitable tissue, the renal epithelial cell line LLC-PK1. As for other MT preparations, we isolated MTs from this cell line that readily formed 2D sheets that generated spontaneous electrical oscillations (Cantero et al., 2016; Gutierrez et al., 2021). This electrical activity was also observed in the primary cilium of the renal epithelial cells. However, the axoneme generated endogenous electrical oscillations different from those elicited by the cytoplasmic MTs, suggesting that the particular arrangement of axonemal MTs produces a frame of electrical oscillators that behaves as an electrical antenna. The present manuscript is arranged in the following sequence (see Figure 1A). We first describe the technique to isolate and purify cytoplasmic MTs and primary cilia from confluent monolayers of LLC-PK1 cells and show the electrical activity of 2D sheets of cytoplasmic MTs. We then present patch clamping data of the ciliary membrane from isolated primary cilia with procedures previously reported (Raychowdhury et al., 2005), where we obtained electrical recordings consistent with spontaneous ciliary ion channels. Subsequently, we permeabilized the primary cilia with Triton-X to access the axoneme and detect electrical oscillations. Finally, we approached the primary cilium under conditions *in situ* and after permeabilization of their membrane, where we also obtained electrical recordings consistent with electrical oscillations. We conducted Time-Frequency and coherence analyses to detect waveform differences between the cytoplasmic and axonemal MTs. Finally, we observed that electrical oscillations are modified in PC2 siRNA depleted cells, linking MT electrical oscillations to ciliopathies. The data indicate that axonemal MTs have distinct electrical properties than cytoplasmic MTs, which may convey the primary cilium’s ability to behave as an electrical antenna, which may explain the universal properties of these organelles.

## Results

### Diagram of experiments and electron microscopy

**FIGURE 2:**
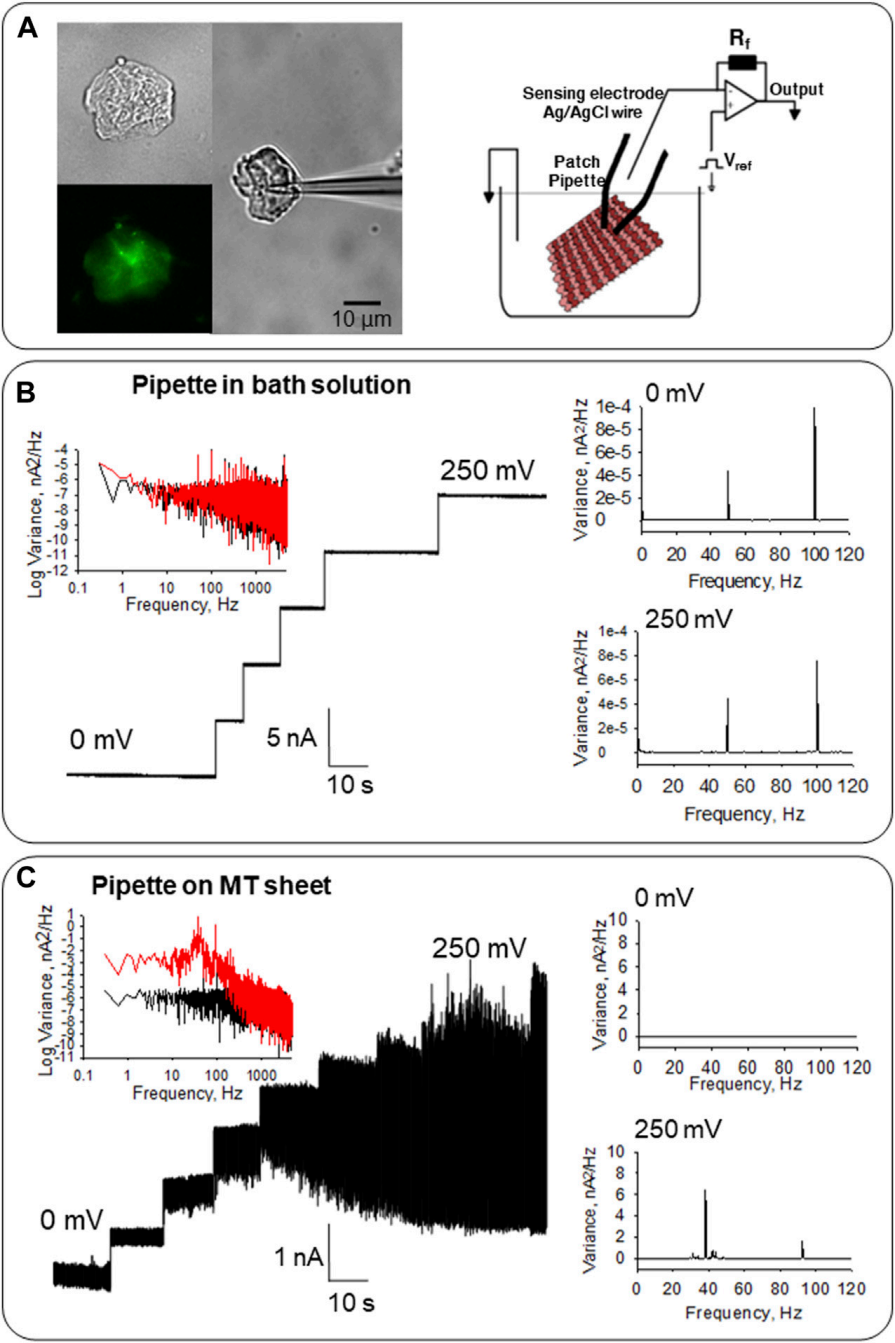
Electrical activity of MT sheets obtained from confluent monolayers of LLC-PK1 cells. **(A)** Left, isolated MT sheet of LLC-PK1 cells in DIC (40x), immunolabeled with anti-α-tubulin (40X) and an attached patch pipette to get electrical signals. Right, the configuration used to obtain electrical signals from MT sheets is described in Cantero et al. (2016). **(B)** Electrical properties of the pipette in solution before MT sheet attachment. Left, the tip pipette in the solution displays clean tracings between 0 and 250 mV. The Fourier spectra show an almost flat response between 10^−1^ and 10^3^ Hz, with a slight 1/f component (Black, 0 mV, Red 250 mV). Right, linear-linear Fourier spectra with only the line contamination peak at 50 Hz, and the first harmonic at 100 Hz at 0 and 250 mV. **(C)** Electrical response of MT sheets. Left, the electrical activity of the same pipette in **(B)** after attachment to a sheet of MTs obtained from LLC-PK1 cells. Oscillations are observed in the entire range. The spectrum at 0 mV (Black) and 250 mV (Red) are shown. Right, the linear-linear Fourier spectra indicate the ∼39 Hz and ∼93 Hz fundamental frequencies of the oscillations in the MT sheet, which are not observed at 0 mV.

In the present study, we explored possible functional differences between the electrical behavior of cytoplasmic MTs and those of the axoneme of primary cilia. For this, we first obtained confluent monolayers of LLC-PK1 cells to isolate and purify cytoplasmic MTs, to make 2D MT sheets as reported (Cantero et al., 2016) (Figure 2A). We also separated primary cilia with procedures previously reported (Raychowdhury et al., 2005) and patched the ciliary membrane. We further permeabilized the isolated primary cilia to obtain electrical data from the axoneme and used Triton X-permeabilized intact cells to obtain electrical recordings of primary cilia *in situ*. We finally conducted Time-Frequency and coherence analyses to detect waveform differences between the cytoplasmic and axonemal MTs.

**FIGURE 3:**
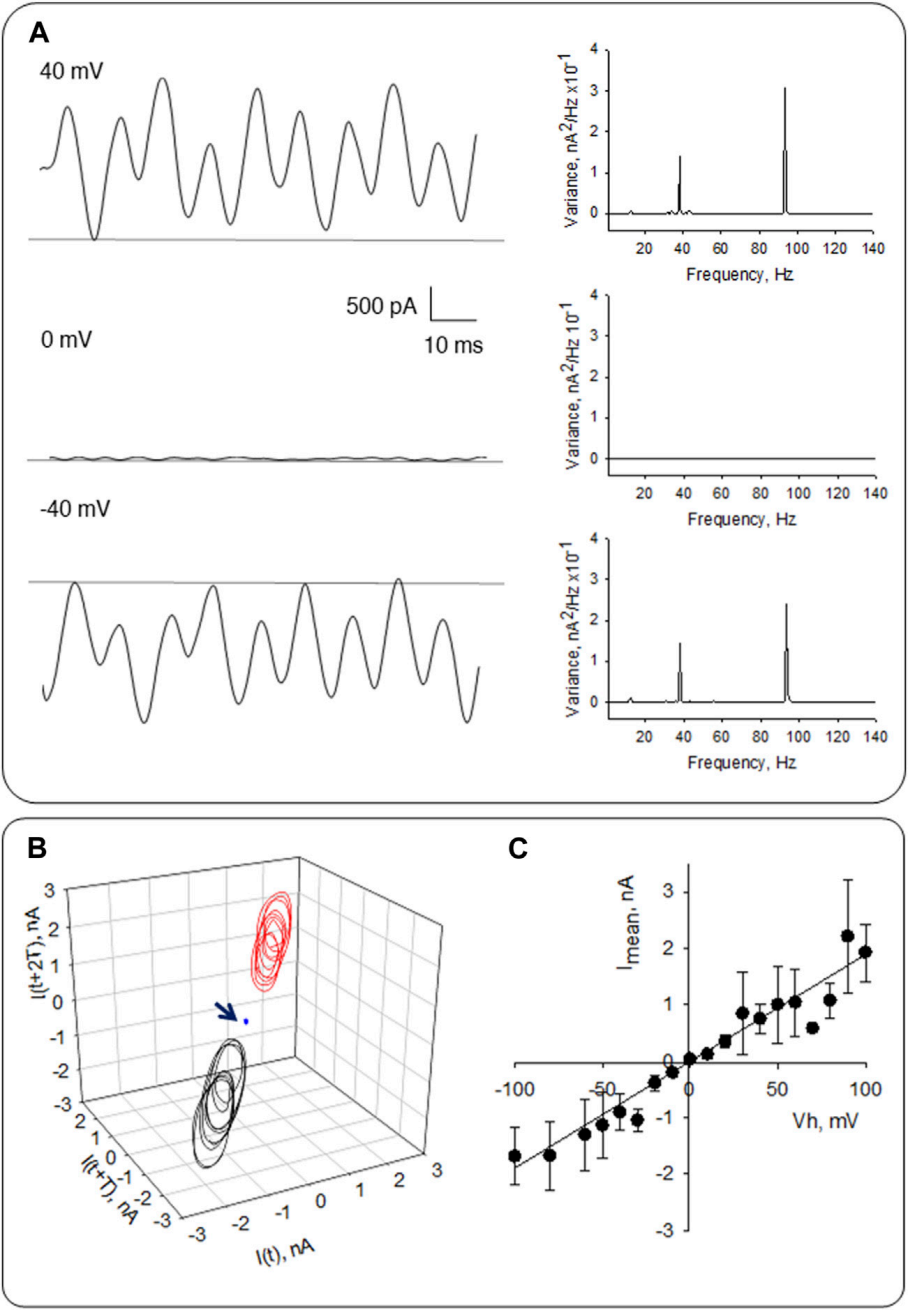
Electrical oscillations of MT sheets from LLC-PK1 cells. **(A)** Left. Electrical oscillations of MT sheets follow the magnitude of the holding potential. Recordings are shown at different applied voltages from the patch pipette. Oscillations are absent at 0 mV under symmetrical KCl. Right. Fourier spectra show fundamental frequencies of ∼39 Hz and 91–93 Hz. **(B)** 3D phase space portrait showing limit cycles at 40 mV (Red), 0 mV (Blue), and −40 mV (Black), respectively. Arrow indicates the plot at zero potential. **(C)** Mean current-to-voltage relationship fitted with the Goldman-Hodgkin-Katz (GHK) equation in symmetrical K^+^ conditions. Experimental values (black circles) were best fitted to a solid line with a slope conductance of 19.1 ± 1.1 nS (r^2^ = 0.9341), for *n* = 6 experiments.

MTs were extracted from confluent monolayers of LLC-PK1 cells, following Fourest-Lieuvin (2006) method, with several modifications (see Materials and Methods, Figure 1A) to test whether MTs from renal epithelial cells generated electrical signals. Confluent monolayers of LLC-PK1 cells were used to isolate and purify cytoplasmic MTs, and obtain 2D sheets (Figures 1B–D). To detect the presence of MTs and MT sheets, we conducted transmission electron microscopy (TEM) on the isolated MT preparation incubated in a high KCl solution. Negative staining of MT sheets rendered images of both MTs and sheets, where the gray scale can help identify the height of the sheets stacked on top of each other. The thickness of the longer MTs (several µm) is about 25 nm. However, the incubation conditions are more prone to produce flat MT sheets, where we could also identify individual protofilaments, including smaller MT-like structures (Figures 1D, 1–3). Small MT sheets showed parallel stacks of protofilaments thinner than the expected MT size.

### Electrical signals from MTs of LLC-PK1 renal epithelial cells

MT sheets were voltage-clamped under “symmetrical” conditions in the presence of an intracellular-like, KCl-containing (140 mM) solution in both bath and patch pipette (Figure 2A, Right). MT sheets were readily observed under DIC microscopy and immuno-labeled with an anti-α-tubulin antibody (Figure 2A, Left). Before attachment to the MT sheet, the pipette floating on bath solution did not show either an electrical response or electrical oscillations, evidencing only the characteristic peaks of line frequency (50 Hz and its corresponding first harmonic, in 100 Hz, Figure 2B). As previously reported, this control condition was performed before the MT sheet was approached with a patch pipette connected to a patch-clamp amplifier, always with the same result (Cantero et al., 2016). After pipette attachment to the MT sheet, spontaneous electrical oscillations were observed at different holding potentials except for 0 mV, as expected, without an electrochemical driving force (*n =* 32, Figures 2C, 3, Middle).

Electrical oscillations varied relatively linearly with the holding potential. However, the seal resistance (lack thereof) also affected the magnitude of the electrical signals. We did not attempt correcting the extent of the amplitude by the actual holding potential at the tip, which is not necessarily the same as the applied voltage. The signals should be considered qualitative representations of the oscillatory behavior.

Frequency-domain analysis of the recordings showed a distinct pattern of discrete frequencies representing the most prevalent oscillatory modes, with peaks at ∼39 Hz and ∼93 Hz (Figure 3A, Right). Most evident were monoperiodic limit cycles observed in 3D phase space Poincaré portraits at 40 mV and −40 mV, but not at 0 mV (Figure 3B), as expected. The mean current-to-voltage relationship was fitted with the Goldman-Hodgkin-Katz (GHK) constant field equation (Schultz, 1980), as a linear conductance of 19.1 ± 1.1 nS (*r* ^2^ = 0.9341, *n* = 6, Figure 3C) in symmetrical KCl. This finding is consistent with its electrodiffusional through the wall of the MT assembly, as previously reported in MT sheets from other origins (Cantero et al., 2016).

### Electrical activity of primary cilia from LLC-PK1 Cells

**FIGURE 4:**
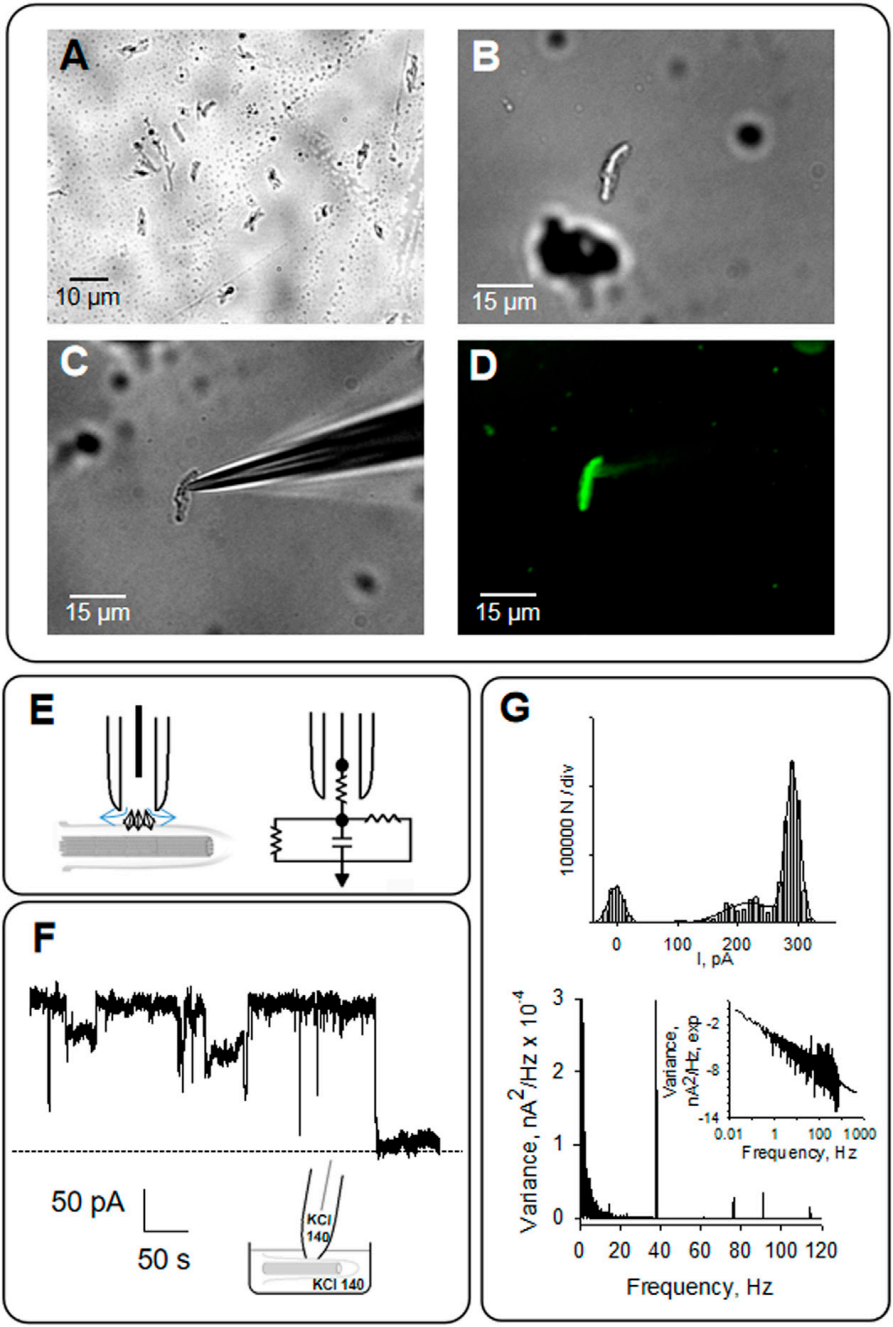
Patch clamping of isolated primary cilia from LLC-PK1 cells. **(A)** Primary cilia obtained as described in Materials and Methods was observed by microscopy (40X). **(B)** Isolated primary cilium was observed under DIC and patched clamped under the loose patch configuration **(C)**, as previously reported (Raychowdhury et al., 2005). **(D)** Primary cilia were identified by immunolabeling with anti-acetylated -α- tubulin antibody exposing the MTs axoneme (40X). **(E)** Schematics of loose patch configuration of the primary cilium. The diamonds represent ion channels at the ciliary membrane. The arrows represent the leak currents at the seal, depicted as vertical and horizontal resistors connected to the ground in the electrical circuit. **(F)** Representative tracing of electrical activity shows ion channels in the presence of symmetrical KCl at 60 mV. **(G)** Top, Amplitude histogram of single-channel tracings, Bottom, frequency domain spectra for tracings in **(F)**. All tracings were obtained in symmetrical KCl.

**FIGURE 5:**
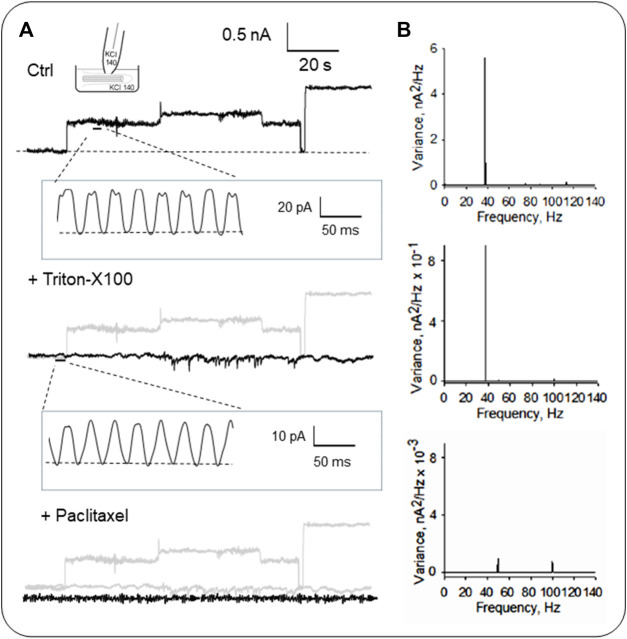
Electrical activity of isolated primary cilia from LLC-PK1 cells. **(A)** Representative single-channel currents of membrane-attached patches under control (Top), and after subsequent addition of the detergent Triton X (Middle) and the MT stabilizer paclitaxel (Bottom) in symmetrical KCl solution at 60 mV. Please note that all panels include a gray tracing representing the original control current, for comparison. The recording’s open-state noise displayed oscillatory behavior (*n* = 6). **(B)** Fourier spectra of the tracings in **(A)** show a fundamental frequency of ∼39 Hz (Top and Middle). Paclitaxel inhibited the MT-driven oscillations, and the spectrum showed two minor peaks at 50 Hz and 100 Hz from line contamination.

As previously described, the organelle was isolated from confluent monolayers (Raychowdhury et al., 2005) to evaluate the electrical activity from the axoneme of LLC-PK1 renal epithelial cell primary cilia. Primary cilia were observed under DIC and immunolabeled with an anti-acetylated-α-tubulin antibody to visualize the axoneme (Figures 4A, B, D). Primary cilia were then patch-clamped using the loose patch configuration (Cantero et al., 2018) under symmetrical ionic conditions described in Materials and Methods (Figures 4C–E). Spontaneous single-channel activity was observed in *n* = 15/25 experiments (Figures 4F, G). Figure 5 is an extension of Figure 4, which details how the electrical oscillations of the axoneme were identified. As previously published, we identified clusters of ion channel currents in isolated primary cilia of LLC-PK1 cells (Raychowdhury et al., 2005; Raychowdhury et al., 2009). To evaluate the presence of electrical oscillations in the axoneme of the primary cilia, the organelles were first loose-patched, and then membrane-permeabilized by addition of Triton X (0.1%, Figure 5). Under these conditions, the channel current deflections disappeared, but disclosed oscillatory currents of the underlying axoneme (Figure 5A, Middle and Bottom tracings). Notably, “buried” in the noise of the open state of the control channel currents as large as 800 pA in amplitude (Figure 5A, Top) also appeared an oscillatory component of ∼40 pA. Thus, the electrical oscillatory component of the cytoskeleton is present in the channel currents, a phenomenon that is likely of interest for future studies of channel-containing membrane patches. Interestingly, the periodic oscillations included in the open state noise of the recordings were confirmed by frequency domain analysis with Fourier transformation of the time series (Figure 5B). The oscillatory signals had a dominant frequency of ∼39 Hz. The results suggested that the oscillations were associated with the ciliary MTs transmitting electrical information from and to the ciliary membrane, which was confirmed by the addition of the MT stabilizer paclitaxel (2.5 μM) that eliminated the axonemal oscillations (Figures 5A, B, Bottom). This agrees with its inhibitory effect on the oscillations in brain MT 2D-sheets and bundles (Cantero et al., 2016; Cantero et al., 2018).

### Electrical activity of *in situ* primary cilia

**FIGURE 6:**
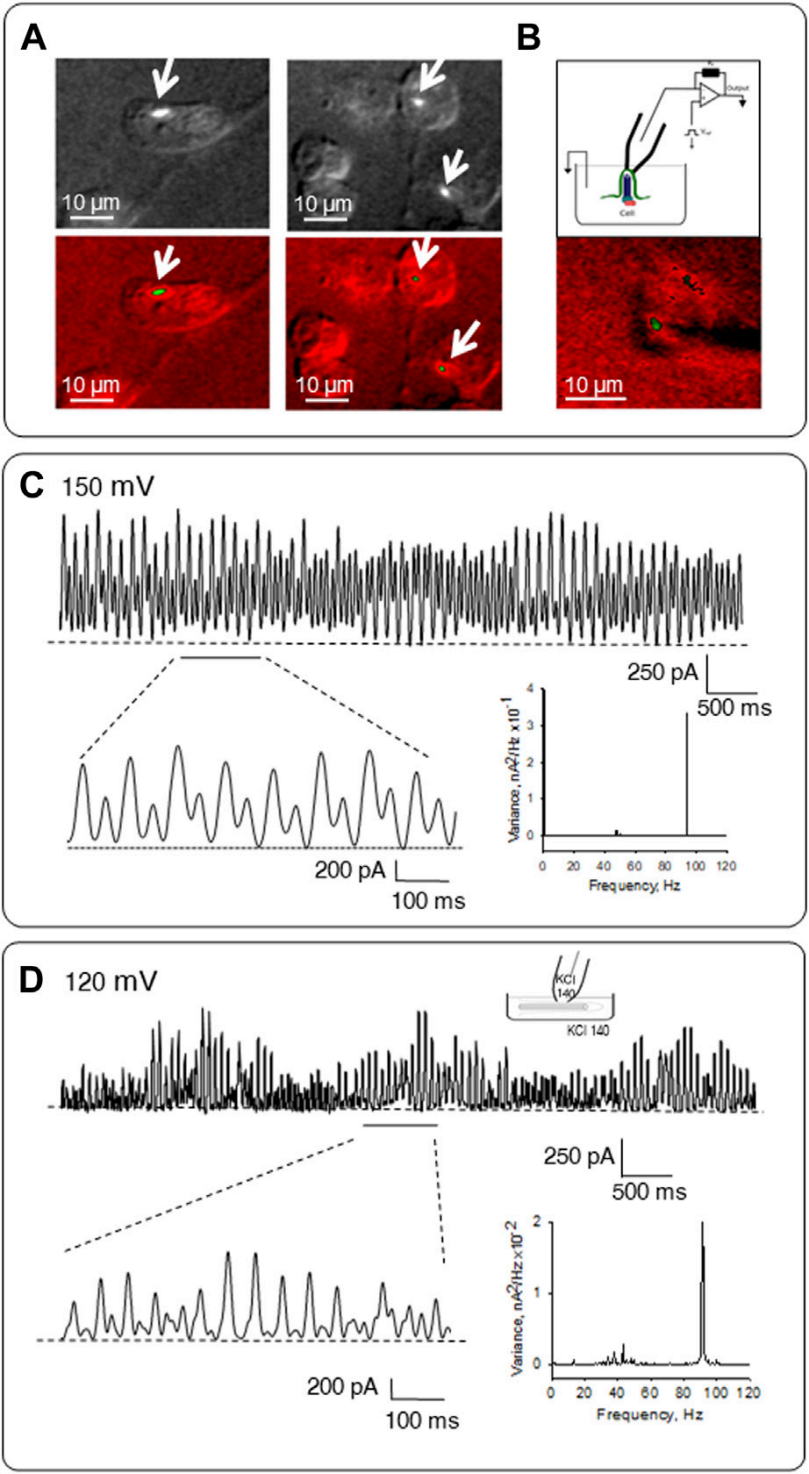
Patch clamping of permeabilized *in situ* and isolated primary cilia. **(A)** Representative images of LLC-PK1 cells incubated in an intracellular-type solution containing high KCl and anti-α-acetylated tubulin antibody complexes to identify the primary cilium. **(B)** Top, Configuration used to obtain electrical signals from MT sheets as described in Cantero et al. (2016). Bottom, Combined DIC-immunofluorescence imaging of an *in situ* primary cilia and the approaching patch pipette. **(C)** Electrical oscillations of *in situ* patched permeabilized primary cilium. Expanded tracings are shown in Bottom Left. Shown tracings were obtained at 150 mV. The frequency-domain analysis of tracings is shown on Bottom Right. **(D)** Electrical activity of permeabilized isolated primary cilia. During overnight incubation, primary cilia were permeabilized in a high KCl solution containing Triton X (0.1%). Top, Representative tracing showing electrical oscillations obtained under symmetrical 140 mM KCl solution conditions. Data representative of *n* = 6. Bottom left, the expanded tracing shows the pattern of electrical oscillations. Bottom right, the frequency spectrum shows a prominent peak at ∼93 Hz and several minor peaks between 29 and 49 Hz. Data were obtained at 120 mV.

The electrical oscillatory activity of the axoneme was also determined in patched permeabilized primary cilia under *in situ* conditions. For these experiments, confluent monolayers of LLC-PK1 cells were incubated in an intracellular-type solution containing high KCl (140 mM, see Materials and Methods), supplemented with both Triton X (0.1%) to permeabilize the cell membranes, and a complex comprising the anti-acetylated α-tubulin antibody, and a FITC fluorescent secondary antibody to label the axoneme. Combined DIC-immunofluorescent imaging was used to identify primary cilium-expressing single cells (Figure 6A), which were then patch clamped under the loose patch configuration (Cantero et al., 2018) (Figure 6B). Electrical oscillations were readily observed from the *in situ* permeabilized primary cilium (*n =* 6, Figure 6C). However, a frequency-domain analysis of the oscillatory tracings indicated that the fundamental frequency differed from the cellular MT sheets and isolated primary cilia. Namely, a most prominent ∼93 Hz fundamental frequency and frequencies of ∼40–49 Hz were observed.

### Electrical activity of permeabilized isolated primary cilia

To better access the ciliary axoneme, the organelle was incubated for 18 h with Triton X (0.1%) to guarantee complete membrane permeabilization. In symmetrical KCl (140 mM) presence, spontaneous single-channel activity was not observed; instead, electrical oscillations were readily present (*n =* 14, Figures 6D). A frequency-domain analysis Figure 6D (Bottom right) showed a pattern of oscillations with distinct frequencies, including a most prominent ∼93 Hz signal and a 29–49 Hz pattern. However, the amplitude of the oscillations followed a linear response concerning the magnitude and polarity of the holding potential in the range of ±80 mV (data not shown), indicating their electrodiffusional nature.

### Time-frequency domain analysis of the electrical oscillations

The frequency-domain analysis by Fourier transformation of the electrical recordings indicated the oscillatory behavior of the tracings that disclosed the prevalent frequency peaks in each preparation. However, high precision in the frequency domain gives no information about the correlation in time. Thus, we applied dual Time-Frequency (TF) algorithms to compare the oscillatory currents from different origins, including the MT sheets (cytoplasmic MTs) and permeabilized primary cilia (axonemal MTs).

**FIGURE 7:**
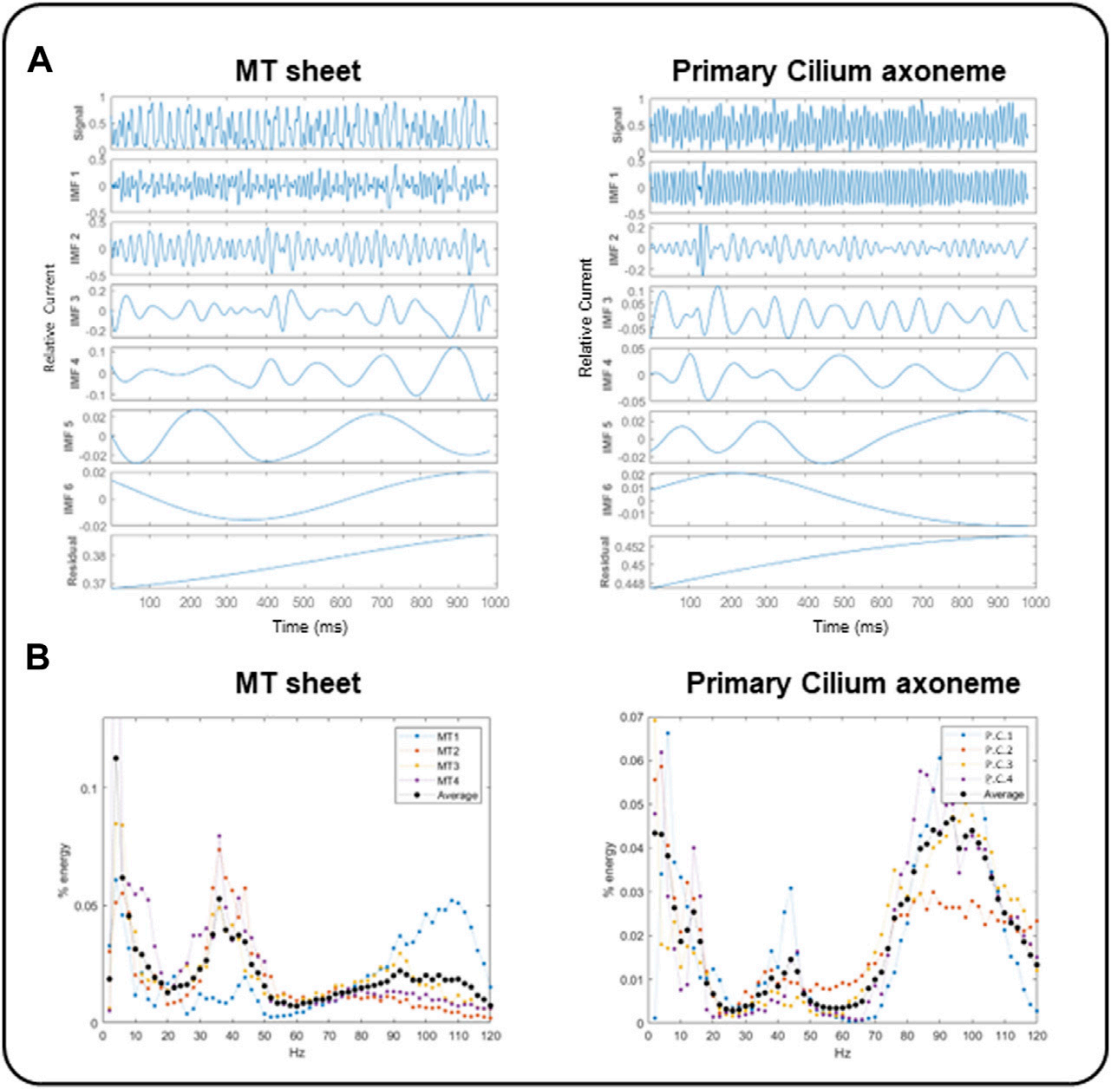
EMD and energy distribution of electrical oscillations from cytoplasmic and axonemal MTs. **(A)** EMD examples of electrical oscillations from LLC-PK1 MT sheets (Left) and permeabilized primary cilia (Right), showing 6 IMF per sample. **(B)** Energy distribution of LLC-PK1 MT sheet currents (Left) and permeabilized primary cilia currents (Right), for *n* = 4 recordings (colored dots), and the average energy (black dots). LLC-PK1 MT sheets evidence a fundamental energy peak in the 30–50 Hz range and a secondary shoulder at 90–110 Hz. The primary cilia axoneme presented its actual energy peak in 80–110 Hz and a smaller one in the range of 30–50 Hz.

We first applied the Empirical Mode Decomposition (EMD), as recently described (Scarinci et al., 2023) to split the original signals into monoperiodic Intrinsic Mode functions (IMFs). MT sheets showed 6 or 7 IMFs while the primary cilia axoneme was decomposed into 6 (Figure 7A), with similar mean frequencies (Table 1, *n =* 4). However, the percentage of energy implied on each function differed in each sample. The total and reduced area under the curve (TAUC and RAUC, respectively) of the LLC-PK1 MT sheets in the range of 32–44 Hz was 15.82% ± 5.82% for TAUC and 21.21% ± 8.01% for RAUC, and 8.18% ± 3.24% and 10.94% ± 4.24% for the 89–96 Hz range, respectively. Conversely, the primary cilium axoneme exhibits a lower percentage in the 32–44 Hz range (7.46% ± 2.94% for TAUC and 9.47% ± 3.04% for RAUC) but a higher value in the 89–96 Hz range (18.86 ± 1.94 for TAUC and 24.47 ± 2.66 for RAUC), demonstrating different energy implied in each frequency range (Table 2). These differences were statistically significant (*p* < 0.05). The energy distribution graph evidences these results (Figure 7B).

**TABLE 1:** Mean frequency value for IMF.

	Cytoplasmatic MT sheet			Primary cilium axoneme		
IMFs	f, Hz	SD, Hz	n	f, Hz	SD, Hz	n
IMF1	90.63	1.43	4	92.00	0.16	4
IMF2	37.72	0.24	4	39.90	4.91	4
IMF3	18.40	5.14	4	13.25	0.17	4
IMF4	4.64	1.30	4	5.13	2.86	4
IMF5	2.65	0.84	4	1.77	1.06	4
IMF6	1.35	0.60	4	1.24	0.87	4
IMF7	0.76	0.11	4	-	-	-

**TABLE 2:** AUC for relative current. *t*-test was performed between samples. *p < 0.05 vs*. MT sheet*.

	Cytoplasmatic MT sheet						Primary cilium axoneme					
Range	%TAUC	SEM	n	%RAUC	SEM	n	%TAUC	SEM	n	%RAUC	SE	n
<2 Hz	7.09	0.96	4	9.45	1.17	4	12.00	0.74	4	15.48*	1.23	4
2–7 Hz	2.83	0.66	4	3.76	0.86	4	1.30	0.37	4	1.68	1.23	4
9–12 Hz	1.04	0.30	4	1.37	0.39	4	0.81	0.11	4	1.04	2.09	4
16–19 Hz	0.95	0.15	4	1.28	0.21	4	0.92	0.21	4	1.17	0.37	4
32–44 Hz	15.82	2.91	4	21.21	4.00	4	7.46	1.47	4	9.47*	3.61	4
89–96 Hz	8.18	1.62	4	10.94	2.12	4	18.86	0.97	4	24.47*	0.71	4

**FIGURE 8:**
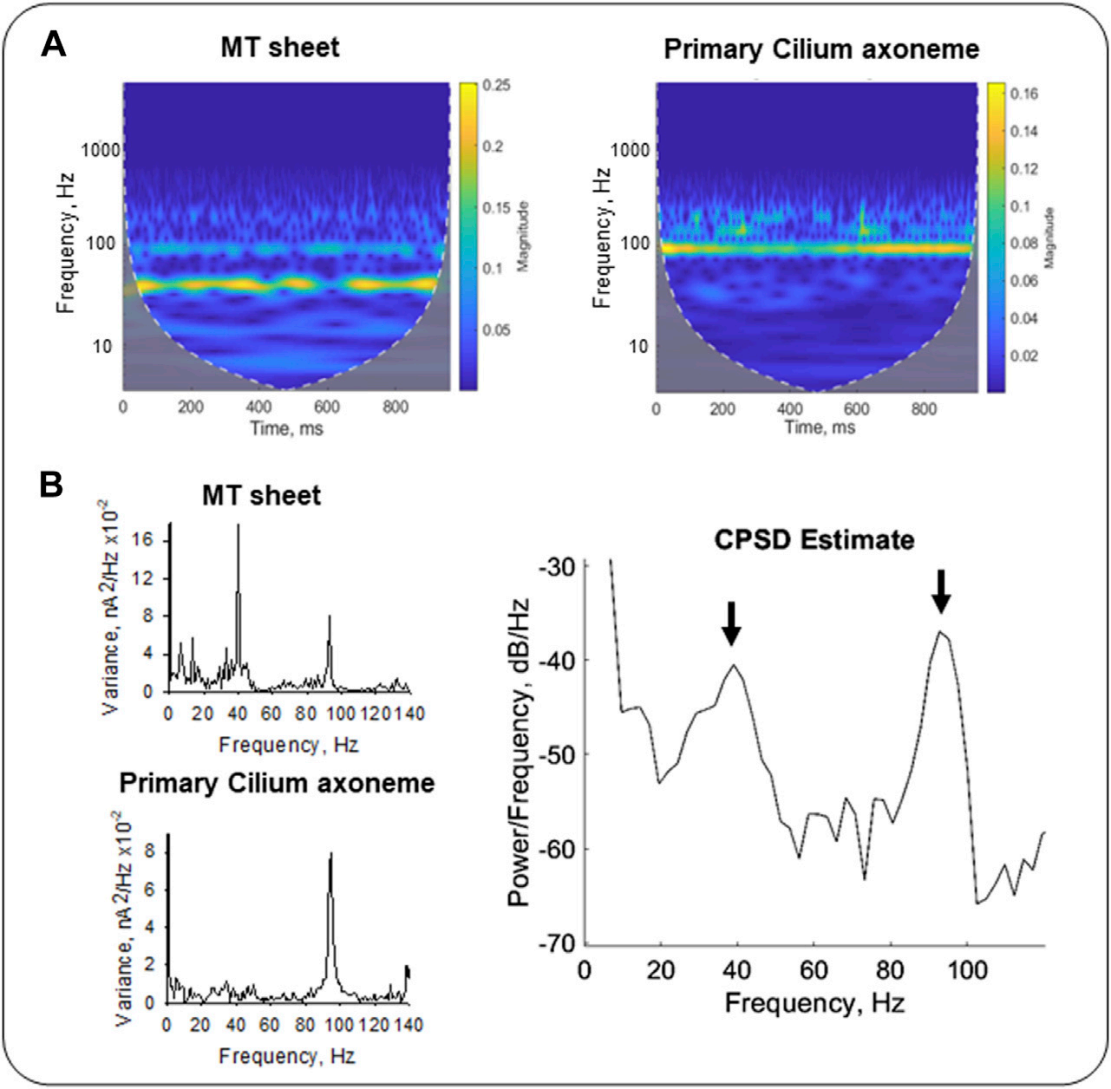
Time-Frequency analysis of electrical oscillations from cytoplasmic and axonemal MTs. **(A)** CWT of electrical oscillations from LLC-PK1 MT sheets (Left) and permeabilized primary cilia (Right). Signals at 40 mV were averaged (*n* = 3 for each sample). White dashed lines draw the cone of influence, showing (Gray) the areas in the scalograms potentially affected by boundary effects. **(B)** Fourier power spectra of LLC-PK1 MT sheet currents (Top Left) and primary cilia currents (Bottom Left). CPSD between both signals is shown (Right). Arrows indicate coherence in predominant frequencies at ∼39 Hz and ∼93 Hz.

To further explore the TF domain, the Continuous Wavelet Transform (CWT) and Cross-Spectral Density (CSD) analyses provided dual TF information of the signals from the wavelet coefficients after decomposition into elementary waveforms (see Materials and Methods, Graps, 1995). The results rendered information about frequency variations through time in our samples. We compared the CWT spectra of LLC-PK1 MT sheets and permeabilized primary cilia (Figure 8A) from averaged signals (see Materials and Methods, *n* = 3). The CWT of the primary ciliary data showed an 87–99 Hz frequency band (Figure 8A Right, Figure 8B Left Bottom), while the MT sheets showed a 35–44 Hz band and only a smoother one of 87–99 Hz (Figure 8A, Left, Figure 8B Top Left). We also applied the cross-spectral density function (CSDF) to define the correlation between the amplitudes of the spectral frequency components. It was developed as the Fourier spectrum of the mutual coherence function (Sharma et al., 2005). The CSDF of the axoneme MTs and MT sheets signals showed coherence between their fundamental frequencies. A notable coherence was observed at the frequency of ∼93 Hz, but much lower albeit significant at 39 Hz (Figure 8B, Right). The processed data indicated that the different MT assemblies share frequency peaks but in different proportions, suggesting a higher degree of coherence or synchrony in the primary cilium, which may correlate with its function in the cell.

### Electrical activity of *in situ* primary cilia on PKD2 silenced cells

**FIGURE 9:**
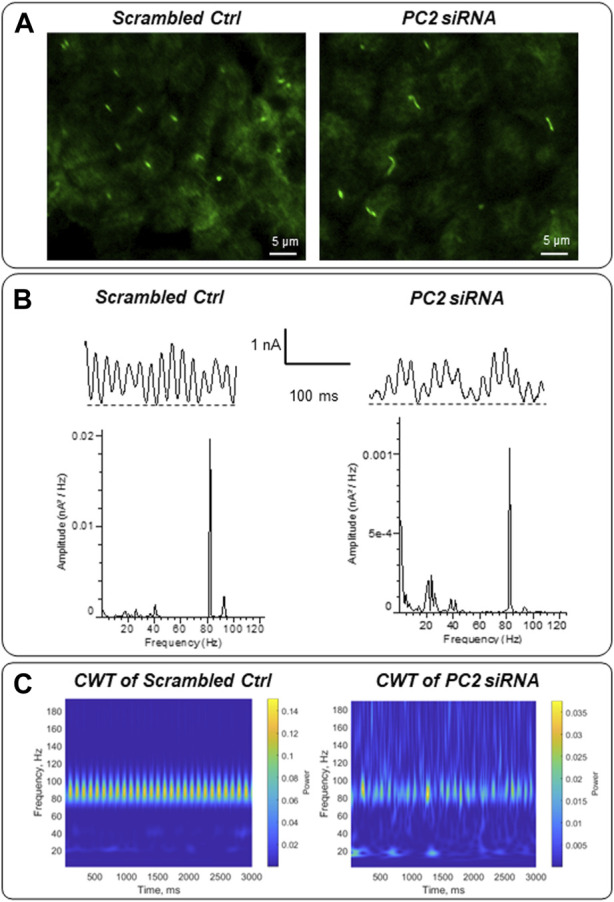
Electrical oscillations of *in situ* primary cilia of PKD2-silenced LLC-PK1 cells. **(A)** Immunofluorescence of LLC-PK1 cells transfected with scrambled (irrelevant) siRNA or PC2 siRNA. Cells were exposed to anti-acetylated-α-tubulin primary antibody and further incubation with FITC coupled anti-mouse IgG. Primary cilia of PC2 silenced cells were longer compared with their controls, as recently reported (Scarinci et al., 2022). **(B)** Electrical oscillations of *in situ* primary cilia were obtained using loose patch-clamp technique. Tracings are representative of *n* = 3, for each condition. The transfection method modifies the predominant frequencies observed in control cells (Figure 6). However, PC2 silenced cells showed a different pattern of 20–25 Hz and 91–93 Hz frequencies. **(C)** The frequency differences can be more effectively observed within a graphical representation of the Continuous Wavelet Transform (CWT), encompassing both the energetic and temporal domains.

Mutations in the PKD2 gene, encoding the TRP channel polycystin-2 (González-Perrett et al., 2001) cause autosomal dominant polycystic kidney disease (ADPKD). To explore whether the electrical activity of the primary cilium may be linked to ciliopathies, LLC-PK1 cells were transfected with siRNA of the PKD2 gene (siPKD2) as recently reported, which inhibits expression of the polycystin-2 (PC2) channel, and lengthens primary cilia in these cells (Scarinci, et al., 2022). Control cells were transfected with an irrelevant probe (*Irss*, scrambled RNA). *In situ* loose patch-clamp experiments were carried out under KCl 140 mM symmetrical conditions (*n =* 3). Fourier power spectra evidences that the transfection method induces changes in the predominant frequencies. However, the difference between the electrical signals of scrambled *Irss* and siPDK2 is remarkable, showing a frequency pattern of 20–25 Hz and a decrease in the 91–93 Hz frequencies in PC2 silenced cells (Figure 9B). The differences are more evident in the CWT graphs (Figure 9C). Interestingly, the presence of lower frequency peaks in the longer (siPKD2) primary cilia is consistent with the behavior of electrical antennas, which largely depend on their physical parameters to control performance (Orfanidis, 2016).

## Discussion

Primary cilia are transducers of environmental signals that regulate cell proliferation, differentiation, transcription, migration, polarity, and survival (Lee and Gleeson, 2010). Dysfunctional ciliary proteins are implicated in human diseases known as ciliopathies. Dysfunctional cystin (Hou et al., 2002) and polaris, located in the ciliary basal body and axoneme (Yoder et al., 2002), and the ciliary channel/receptor complex proteins polycystins 1 (PC1) and 2 (PC2), which cause ADPKD, generate ciliopathies (Murcia et al., 2000; Hou et al., 2002; Pazour et al., 2002; Yoder et al., 2002). The various models of cystic disease established a connection between primary cilia and MTs (Ma et al., 2017). *Orpk* mice with dysfunctional polaris have shortened cilia, left-right symmetry defects (Murcia et al., 2000), and more abundant PC2 in the primary cilia (Pazour et al., 2002). Also, homozygous *cpk/cpk* mice with cystic kidney disease moderate their progression by treatment with the microtubular stabilizer paclitaxel (Woo et al., 1997). Therefore, in morphological terms, the axoneme-regulated ciliary structure is linked to maneuvers that modulate the primary cilium length (Scarinci et al., 2020; Scarinci et al., 2022). Also, there is strong evidence for the electrical activity of the renal primary cilium. Previous studies obtained direct electrical information on the ciliary membrane, isolated (Raychowdhury et al., 2005) and recently *in situ* (Kleene and Kleene, 2012; DeCaen et al., 2013; Delling et al., 2013). However, how ciliary channel activity translates into cell signaling and activation remains an open question.

MTs are bio-electrochemical transistors acting as nonlinear transmission lines capable of generating electrical oscillations and transmitting electrical signals at a distance (Cantero et al., 2018; Gutierrez et al., 2020). MTs produce time-dependent, frequency-modulated, highly synchronized substantial changes in ionic conductance triggered by their gating mechanism as memristive devices (Cantero et al., 2019; Vissol-Gaudin et al., 2021). To date, there is no available information on the role of MTs comprising the axonemal structure of the primary cilium on the electrical activity of the organelle.

In the present study, we demonstrated that cytoplasmic MTs from LLC-PK1 renal epithelial cells produce robust electrical oscillations that resemble the oscillatory behavior of brain MTs (Cantero et al., 2018; Gutierrez et al., 2020; Gutierrez et al., 2023) and the cytoskeleton of permeabilized cultured mouse hippocampal neurons (Cantero et al., 2019; Cantero et al., 2020; Gutierrez et al., 2020). This electrical activity may couple to, or regulate, the activity of membrane events, including ion channel activity, and *vice versa*. Here we observed that the MTs of the axoneme were similar in their electrical behavior to that of cytoplasmic MTs while connected to the ciliary membrane. Soon after permeabilization, their behavior shifted with a distinct pattern of fundamental frequencies that further changed after overnight permeabilization and incubation in an intracellular-type solution. Because each MT is an entirely functional electrical oscillator (Cantero and Cantiello, 2020), the geometry of nine doublets observed in the axoneme is necessarily richer and more complex. Its distinct electrical properties will depend on its specific MT assembly.

We observed that the primary cilia from LLC-PK1 cells evidenced spontaneous ion channel activity, as previously reported (Raychowdhury et al., 2005; Raychowdhury et al., 2009). However, a more detailed inspection of the open-state noise of the currents showed periodic oscillations with a ∼39 Hz frequency, similar to that observed in MTs. While ion channel activity disappeared after ciliary membrane permeabilization, the electrical oscillations were more evident. The electrical activity of the *in situ* permeabilized primary cilia evidenced fundamental frequencies that differed from the cytoplasmic MTs and the isolated primary cilia. Namely, we observed a prominent ∼93 Hz fundamental frequency not apparent in mammalian brain MT preparations (Cantero et al., 2016; Cantero et al., 2018) but previously reported in honeybee brain MT preparations (Gutierrez et al., 2021), resembling high gamma brainwaves (Miltner et al., 1999).

The dual Time-Frequency (TF) algorithms we used to compare the oscillatory currents from the different preparations (Figure 1), including the cytoplasmic MTs and the permeabilized primary cilia, confirmed the different oscillatory regimes. The EMD (Scarinci et al., 2023) showed that the percent energy implied differed in each frequency range. The CWT and CSD were applied and further confirmed these findings. The CWT of the primary ciliary data showed an 87–99 Hz frequency band that was smoothened in the cytoplasmic MTs, which additionally led to a more prominent 35–44 Hz band. The cross-spectral density function (CSDF) of the axoneme and cytoplasmic MT signals also showed coherence between their fundamental frequencies, most notably at the frequency of ∼93 Hz. Thus, the different MT assemblies would share frequency peaks but in different proportions, suggesting a higher degree of coherence or synchrony in the primary cilium, which may correlate with its function in the cell.

Concerning the axonemal and cytoplasmic MTs, posttranslational modifications (PTM) and the different tubulin isotypes may play a role in their differences in electrical activity. However, different preparations of brain microtubules, including those heavily doped with MAPs, compared with purified commercial tubulin (Cantero et al., 2016; Gutierrez et al., 2023) showed highly similar frequency domain responses. Thus, the hypothesis is forwarded that the arrangement’s specific geometry in the axoneme may be critical to this signal discrimination (Cantero et al., 2016; Cantero et al., 2018; Gutierrez et al., 2021), and the present results support this idea because of the unique MT disposition of the primary cilia axoneme (9 + 0). Further, preliminary evidence also showed that the tubulin ancestor FtsZ, which does not make microtubules but forms sheets and rings, also generates electrical oscillations (Bonacina et al., 2020; Carabajal et al., 2023).

Finally, we observed that a maneuver mimicking an ADKPD triggering signal in PKD2 gene antisense RNA expressing cells affects the electrical oscillations of axonemal MTs. Cells treated with PKD2 antisense RNA disclosed a frequency range not previously observed in either control cells or cells treated with the scrambled probe used as negative controls. Thus, the antisense delivery system also seemed to affect the axonemal MT pattern of electrical oscillations. This information, along with the electrical differences observed between permeabilized and non-permeabilized isolated and *in situ* primary cilia, suggest that ciliary ion channel activity may play a role in the electrical signaling through MTs, since they regulate ion channel activity such as that of PC2 (Montalbetti et al., 2007), for which future studies are required.

In conclusion, the electrical activity of the axoneme in the primary cilium of renal epithelial cells is consistent with an electrical antenna that generates and conducts electrical oscillations of specific frequencies that can be driven to the cell’s interior. As an electrical amplifier, the axoneme is an integral component of an electrodynamic network in conjunction with current generators (i.e., MT-regulated channels, Li et al., 2006) and MT-coupled intracellular transmission lines (F-actin) (Lin and Cantiello, 1993; Tuszynski et al., 2004). Electrical amplification by axonemal MTs may be central to sensing environmental signals by the primary cilium.

We provide evidence for a novel signaling mechanism associated with the sensory function of primary cilia, consistent with the electrical oscillations of MTs, whose frequency shifts may aid our understanding of ciliopathies as ADPKD. Future studies will further explore the resonance and chaotic properties of the oscillating frequencies (Aguiar-Conraria and Soares, 2011) and how they affect cell function.

## Materials and Methods

### Cell culture

The present studies on primary cilia from LLC-PK1 renal epithelial cells (ATTC CL-101) were conducted with previously reported techniques (Raychowdhury et al., 2005; Dai et al., 2017). Briefly, cells were maintained in Dulbecco’s modified Eagle’s medium (DMEM) supplemented with 3% fetal bovine serum (FBS) and grown to confluence in a humid atmosphere of 5% CO_2_ and 37°C, renewing the culture medium once a week.

### Tubulin purification from LLC-PK1 cells

As previously reported, tubulin was obtained from LLC-PK1 cells (Fourest-Lieuvin, 2006) with modifications. Briefly, confluent monolayers grown on Petri dishes in DMEM were rinsed with PBS, trypsinized at 37°C, and supplemented with 3% FBS. The sample was centrifugated at 320*g* for 3 min at 37°C. The pellet obtained was washed with PEM (100 mM PIPES pH 6.7, 1 mM EGTA, 1 mM MgCl_2_) at 37°C and centrifugated at 320*g* for 3 min. The pellet was resuspended for cell lysis in 40 mL of OPT Buffer at 37°C (80 mM PIPES, pH 6.7, 1 mM EGTA, 1 mM MgCl_2_, 0.5% Triton-X, 10% Glycerol, 1 µM pepstatin, 400 µM PMSF). The lysed cells were centrifuged at 320*g* for 3 min at 37°C and carefully discarded in the supernatant. The pellet obtained was resuspended in 2 mL of OPT at 4°C and incubated for 15 min on ice. At the end of this incubation, it was ultracentrifuged at 200,000*g* for 10 min at 4°C. The supernatant, called HOPT extract, was collected. Subsequently, 9 mL of HOPT extracts were supplemented with 5 mM MgCl_2_, 1 mM GTP, and 5% DMSO. The solution was then incubated for 30 min at 35°C to allow polymerization of the MTs. The sample of polymerized MTs was placed on a PEM cushion, 60% glycerol, and 400 µM PMSF at 35°C and ultracentrifuged at 200,000*g* for 20 min at 35°C. The MTs were washed, without resuspension, with 3 mL of PEM50 (35°C, 50 mM PIPES, pH 6.7, 1 mM EGTA, 1 mM MgCl_2_, 1 μM pepstatin, and 400 µM PMSF). Finally, the resulting tubulin suspension was ultracentrifuged at 200,000*g* for 10 min at 4°C, and the supernatant was collected. The samples were aliquoted and stored at −20°C until use.

### Isolation of primary cilia from LLC-PK1 renal epithelial cells

Wild-type LLC-PK1 renal epithelial cells were cultured as previously described (Raychowdhury et al., 2005) in DMEM supplemented with 3% FBS. Cells were seeded onto glass coverslips and grown at 37°C in a humidified atmosphere with 5% CO_2_ to reach full confluence. Confluent cells seeded onto glass coverslips (seven to ten days) were used for immunocytochemical studies. Confluent monolayers were scraped with Ca^2+^-free phosphate-buffered saline and centrifuged for 5 min at 52*g* whenever isolated primary cilia were required. The cell pellet was suspended in a high Ca^2+^ “deciliation” solution containing 112 mM NaCl, 3.4 mM KCl, 10 mM CaCl_2_, 2.4 mM NaHCO_3_, 2 mM HEPES, pH 7.0. Resuspended cells were shaken in this solution for 10 min at 4°C. Isolated primary cilia were separated by centrifugation at 7,700*g* for 5 min. The supernatant was loaded on top of a 45% sucrose solution in a high Ca^2+^ saline solution and centrifuged for 1 h at 100,000*g*. The sucrose-supernatant interface band was collected, diluted (1:10), and centrifuged again for 1 h at 100,000*g*. The pellet was resuspended in a standard saline solution at pH 7.0 and supplemented with 2.0 mM EGTA and 0.5 mM sucrose. Samples were aliquoted and stored at −20°C until further use.

### PKD2 gene silencing

As recently reported, silencing of PKD2 gene expression in cultured LLC-PK1 cells was conducted using the small interfering RNA technique (Dai et al., 2017; Scarinci et al., 2022). Briefly, two 21-nt PKD2-specific synthetic siRNAs, one of which was a fluorescent (fluorescein) probe, were synthesized by Invitrogen (Buenos Aires, Argentina), as well as a 19-nt irrelevant sequence as scrambled control (Ir-siRNA). As reported initially, all constructs bore dTdT overhangs at the 3′end. The siRNAs sense sequences were as follows; (siPKD2) GCU​CCA​GUG​UGU​ACU​ACU​ACA, starting at 906 in exon 3 of the porcine PKD2 gene, and (Ir-siRNA) UUC​UCC​GAA​CGU​GUC​ACG​U, as scrambled control. siRNA transfection was conducted as follows: cell cultures were trypsinized and placed at 70% confluence in 35-mm cell culture dishes containing DMEM supplemented with 3% FBS at 37°C in a 5% CO_2_ atmosphere. The following day, transfection was performed with Lipofectamine 2000 (Invitrogen). Tubes were added either scrambled (Irss, 10 µL) or antisense (siPKD2, 10 µL) RNA with DMEM without FBS (100 μL) in presence of Lipofectamine (2 μL). Briefly, the tubes were incubated for 5 min at room temperature and then mixed with either Irss or siPKD2 for another 20 min (200 μL total volume). Incubation was conducted by medium change with a mixture of fresh medium (800 μL, DMEM plus 3% FBS) and 200 μL of the transfection mixture. The total transfection time was three overnights (72 h). Silencing efficiency was confirmed by the Western blot technique, as previously reported (Dai et al., 2017; Scarinci et al., 2022).

### Electron microscopy-negative staining

Transmission electron microscopy (TEM) was conducted with a Zeiss LIBRA 120 transmission electron microscope (CIME, CONICET-UNT). Briefly, 20 µL of the MT suspension was deposited onto a piece of Parafilm^®^ forming a drop, and a 400-mesh nickel grid with a Formvar carbon film was placed over each drop for 5 min. The samples were then stained for 1 min with 2% aqueous uranyl acetate, removing excess staining from the grids with filter article, and allowed to air dry. The grids were examined immediately afterward.

### Immunochemical labeling of MT structures

Isolated primary cilia were labeled with a mouse monoclonal anti-acetylated-α-tubulin antibody (1:100) (clone 6-11B-1, T7451, Sigma-Aldrich, MO) to visualize the axoneme and, as a secondary antibody, a goat anti-mouse IgG-FITC (1:500, #31569 Invitrogen, MA) was used. The antibody raised from rabbits against amino acids 149–448 human α-tubulin was obtained from Santa Cruz Biotechnology Inc. (H-300, sc-5546) and utilized at 1:500 dilutions (Cantero et al., 2018). The secondary antibody used for tubulin staining was bovine anti-rabbit IgG-FITC (sc-2365, Santa Cruz Biotechnology Inc., CA) used at a 1:1000 dilution. Samples were viewed under an Olympus IX71 inverted microscope connected to a digital CCD camera C4742-80-12AG (Hamamatsu Photonics KK, Bridgewater, NJ). Images were collected with the IPLab Spectrum (Scanalytics, Viena, VA) acquisition and analysis software running on a Dell-NEC personal computer.

### Reagents

Unless otherwise stated, chemical reagents were obtained from Sigma-Aldrich (St. Louis, MO, United States) and diluted to their final concentrations.

### Electrophysiological data acquisition and analysis of ciliary structures

The electronic setup to obtain electrical recordings from permeabilized primary cilia consisted of an E-patch amplifier (Elements, Cesena, Italy) directly apposed to the sample via a saline-containing patch pipette, as previously reported (Cantero et al., 2018; Gutierrez et al., 2021). Experiments were conducted with an “intracellular” solution containing, in mM: KCl 140, NaCl 5, EGTA 1, and HEPES 10, adjusted to pH 7.18 with KOH. Patch pipettes were made from soda-lime capillary tubes (Biocap, Buenos Aires, Argentina) with a 1.25 mm internal diameter and a tip diameter of ∼4 μm. Voltage clamp protocols included gap-free data acquisition at various holding potentials from 0 mV. Electrical signals were acquired, digitized, and stored in a personal computer. Data were analyzed with the software suite pCLAMP 10.7 (Molecular Devices, San Jose, CA, United States). Sigmaplot Version 10.0 (Systat Software Inc., San Jose, CA, United States) was used for statistical analysis and graphics.

### Loose-patch clamp configuration

The loose-patch-clamp design was used to patch the axoneme of permeabilized cilia, as previously reported for MT bundles and hippocampal neurites (Cantero et al., 2018). Loose patches have smaller seal resistances (MΩ range) than tight patches obtained from intact cilia or MT sheets. Significant currents flow through the seal and affect the magnitude of the tip potential (Figure 4E). In our preparations, the command voltage (*V*_*cmd*_), which is the holding potential driven by the patch amplifier, is not the one “sensed” by the tip potential on the surface of the primary cilium. The difference will be inversely proportional to the magnitude of the seal resistance. A simple model circuit analysis was used to obtain the voltage at the pipette tip (*V*_*tip*_), which is given by

$$V_{tip}=V_{cmd}\left(\frac{R_{seal}}{R_{seal}+R_{pip}}\right)=V_{cmd}\times B$$ (1)

*R*_*pip*_, *R*_*seal*_, and *V*_*cmd*_ are the pipette resistance, seal resistance, and command voltages. Under such conditions, where *R*_*seal*_ is of the order of magnitude of *R*_*pip*_, the voltage at the pipette tip will thus be reduced from the command voltage by a factor of *B*, which is the magnitude of the voltage divider resolved by the patch seal resistance (note that when *R*_*seal*_ *>> R*_*pip*_, then *V*_*pip*_ *≈ V*_*cmd*_, as in the tight-seal case).

### Other current analyses

Unless otherwise stated, electrical tracings shown throughout the study were unfiltered data. Average currents at various holding potentials were obtained by integrating 1-s tracings and expressed as mean ± SEM values, where (*n*) represented the number of experiments analyzed for a given condition. Limit cycles were constructed using the unfiltered tracings’ time delay (*τ*) approach. The lag time *τ* was chosen arbitrarily at 2*f*, where *f* was the sampling frequency of data acquisition. Three-dimensional phase space diagrams were constructed in Sigmaplot 10.0 (Systat Software Inc., San Jose, CA, United States).

### Spectral analysis

Fourier power spectra and signal filtration were performed using Clampfit 10.7 (Molecular Devices, San Jose, CA, United States). SigmaPlot 10.0 Software (Systat Software Inc., San Jose, CA, United States) was used as a graphing tool.

### Empirical mode decomposition (EMD) analysis

The EMD method decomposes wavelet signals into monoperiodic intrinsic mode functions (IMFs) and was recently proposed as an adequate approach to quantify the frequencies of MT electrical oscillations (Scarinci et al., 2023). Briefly, 1-s, gap-free electrical recordings were filtered to eliminate noise using a Low-pass Gaussian filter at 200 Hz and a Notch filter of 50 Hz (±3 Hz around the central frequency) to attenuate electrical line noise, using Clampfit 10.7 (Molecular Devices, San Jose, CA, Unites States). Filtered tracings were relativized to a min value of 0 and a max value of 1 using a custom Matlab 2019a Trial Version (MathWorks, Natick, MA, Unites States) function. Preconditioned signals were decomposed using the Matlab “emd” function. Each IMF data raw was fitted using the Matlab “Curve Fitting Toolbox."

### Calculation of relative energy

The Fourier spectrum was used to calculate the areas under the curve (AUC) in the ranges reported before (<2 Hz, 2–7 Hz, 9–12 Hz, 16–19 Hz, 32–44 Hz, 89–96 Hz) for further comparison (Scarinci et al., 2023), to find out the energy involved in each IMF per sample. A Reduced Area Under the Curve (RAUC) with a reduced frequency range was selected at 0–140 Hz, as used in the Spectral Edge Frequency (SEF) in the analysis of electroencephalogram (EEG). The percentage of energy involved in each IMF per frequency peak was also calculated and graphed using each IMF instant frequency and energy with a 2D histogram Matlab custom function.

### Continuous wavelet transform (CWT) analysis of the data

To compare the electrical behavior of LLC-PK1 MT structures from different cell domains, namely the cytoplasm (MT sheets) and the primary cilium (axoneme), we used the CWT (Aguiar-Conraria and Soares, 2011), which maps the original time series as a function of one variable, time, into two variables, time and frequency.

The mother function *ψ* behaves like a wave with a decaying property, which contrary to the Fourier transform, decomposes the signal in terms of sines and cosines. This property provides an effective localization in both time and frequency. Starting with a mother wavelet *ψ*, a family *ψ*_*τ,s*_ of “wavelet daughters” can be obtained by scaling and translating *ψ*, such that:

$$\psi_{t,s}\left(t\right)=\frac{1}{\sqrt{\left|s\right|}}\psi \left(\frac{t-\tau }{s}\right),s,\tau \;\epsilon \;R,s\neq 0,$$ (2)

where *s* is a scaling factor controlling the wavelet’s width, and *τ* is a translation parameter controlling the wavelet’s location. Given a time series *x*(*t*) *ϵ L* ^2^ (**R**), its *continuous wavelet transform* (CWT) concerning the wavelet is a function of two variables, *W*_*x,ψ*_(*τ,s*):

$$W_{x,\psi }\left(\tau ,s\right)=\int_{-\infty }^{\infty }x\left(t\right)\frac{1}{\sqrt{\left|s\right|}}\psi \left(\frac{t-\tau }{s}\right)dt$$ (3)

The (local) wavelet power spectrum or scalogram (periodogram) is defined as

$${\left(WPS\right)}_{x}\left(\tau ,s\right)={\left|W_{x}\left(\tau ,s\right)\right|}^{2}$$ (4)

To describe the time-frequency localization properties of the CWT, we have to assume that both the wavelet ψ (*t*) and its Fourier transform ψ (*ω*) are well-localized functions.

The CWT provides a time-scale representation of the analyzed function, not a time-frequency model.

Matlab software (v 2019a) was used for these analyses and implemented its “*cwt*” function.

### Cross-spectral density (CSD) analysis

The coherence of the electrical oscillations in cytoplasmic and axonemal MTs was also assessed for tandem oscillations or, more precisely, the phase relationship between the signals. The coherence between signals defines the degree of confidence one can predict the amplitude and phase at a point (Sharma et al., 2005). An analytical fluctuating signal *V* (*r*,*t*), where *r* is a position vector at a *t* time, has a mutual coherence at two points (*r*_1_ and *r*_2_) as:

$$\Gamma \left(r_{1},r_{2},\tau \right)\equiv \langle V\times \left(r_{1},t\right)V\left(r_{2},t+\tau \right)\rangle$$ (5)

The mutual coherence function is the Fourier inversion and further integration fo*r t*, which retrieves a Dirac *δ* function, such that:

$$\langle V\times \left(r_{1},v\right)V\left(r_{2},v^{\prime }\right)\rangle =\delta \left(V-v^{\prime }\right)W\left(r_{1},r_{2},v\right)$$ (6)

where

$$W\left(r_{1},r_{2},v\right)=\int_{-\infty }^{\infty }\Gamma \left(r_{1},r_{2},\tau \right)e^{2\pi iv\tau }d\tau$$ (7)

*W* (*r*_1_, *r*_2_, *τ*) is the cross-spectral density function (CSDF), or cross-power spectrum (CPS), at the points *P*_1_ (*r*_1_) and *P*_2_ (*r*_2_). The mutual coherence function (Eq. 5) describes the correlation in the space-time domain, while CSDF also characterizes a measure of the correlations in the space-frequency domain (Mandel and Wolf, 1976). Briefly, CSDF is the Fourier Transform of the autocorrelation and cross-correlation function, the cross-correlation function that defines the relationship between two random signals (Oppenheim et al., 1999).

The above analyses were carried out with the Matlab function “*cpsd*” without windowing, which uses Welch’s averaged, modified periodogram spectral estimation method.

### Statistical analysis and graphic processing

For comparison of energy percentages, *t*-test was performed between ranges (α = 0.05). Normality and equal variance tests were previously conducted using Shapiro-Wilk and Levene, respectively. Average percentages were expressed with mean ± SEM, as the variables followed a Normal distribution. Images were processed using ImageJ software (NIH, United States). The diagrams shown in Figures. 1, 2, 4 were constructed with the free and open-source software Inkscape 1.2.1.

## Data Availability

The raw data supporting the conclusions of this article will be made available by the authors, without undue reservation.
